# Altered microRNA Signatures in Sputum of Patients with Active Pulmonary Tuberculosis

**DOI:** 10.1371/journal.pone.0043184

**Published:** 2012-08-10

**Authors:** Zhengjun Yi, Yurong Fu, Rui Ji, Ruifang Li, Zhiyu Guan

**Affiliations:** 1 Department of Laboratory Medicine, Key Laboratory of Clinical Laboratory Diagnostics in Universities of Shandong (Weifang Medical University), Clinical Faculty (Affiliated Hospital) Weifang Medical University, Weifang, China; 2 Department of Medical Microbiology, Weifang Medical University, Weifang, China; National Institute for Infectious Diseases (L. Spallanzani), Italy

## Abstract

Role of microRNA (miRNA) has been highlighted in pathogen-host interactions recently. At present, their role in active pulmonary tuberculosis is unknown. The aim of the study was to delineate miRNA expression in sputum supernatant of patients with active pulmonary tuberculosis. Expression of miRNAs was evaluated by microarray analysis and differentially expressed miRNAs were validated by RT-qPCR. Secreted cytokines TNF-α and IL-6 were measured by ELISA. We found that 95 miRNAs were differentially expressed between tuberculosis group and controls. More miRNAs (52 out of 95 miRNAs) were underexpressed than overexpressed during tuberculosis infection. Overexpression of miR-3179, miR-147 and underexpression of miR-19b-2* in TB group compared with controls were confirmed in the validation cohort. TNF-α and IL-6 levels were not significantly altered between TB group and controls. For the first time, differential expression of miRNAs in sputum was found in active pulmonary tuberculosis. The study provides rationale for identifying the role of miRNAs in the pathogenesis of pulmonary tuberculosis and indicates potential for miRNA-based therapeutic strategies.

## Introduction

MicroRNAs (MiRNAs) are small, noncoding RNAs (∼22 nt) that have key roles in regulation of many biological processes, such as development and tumorigenesis, via regulating expression of their target mRNAs [1], [2], [3]. Emerging evidence also throws light into the role of miRNAs in the intricate host-pathogen interaction networks [4], [5]. For example, miRNA-155 is essential for the T cell-mediated control of helicobacter pylori infection [6]. Let-7 family members repress the expression of IL-6 and IL-10 during S*almonella* infection [7]. MiR-147 attenuates TLR-induced inflammatory responses [8].

Tuberculosis (TB), caused by *Mycobacterium tuberculosis* (*M. tuberculosis*), is one of the most deadly infectious diseases [9]. To date, the role of miRNAs in the pathogenesis of active pulmonary TB has not yet been elucidated. MiRNA spectrum in body fluids can reflect altered physiological and/or pathological conditions [10]. Recent studies have shown that miRNAs are stably present in sputum [11], [12] and unique miRNA signatures in sputum are altered in many lung diseases, such as lung cancer and chronic obstructive pulmonary disease [13], [14]. Taking into account the central role of miRNAs in disease and significance of sputum in the assessment of lung disease [15], we hypothesized that miRNA expression is altered in sputum of patients with active pulmonary TB. Therefore, the study was aimed to detect miRNAs expression in sputum and to develop further understanding of the role of miRNAs in active pulmonary TB.

## Materials and Methods

### Human subjects

Fifty-eight patients with active pulmonary TB were enrolled from Affiliated Hospital of Weifang Medical University and Weifang Chest Hospital, China, between December 2009 and January 2011. Eligibility for patients entry into the study included typical symptoms of pulmonary TB such as cough, fever, fibrocavitary lung infiltrate on chest radiograph, at least one positive sputum smear and/or positive sputum culture for *M.tuberculosis.* Biochemical tests such as niacin production and nitrate reduction were carried out to identify *M.tuberculosis.* Patients were excluded who had other coexisting lung disease. Thirty-two healthy age and sex matched volunteers were recruited as controls. Tuberculin skin test (TST) was performed to exclude latent TB infection (LTBI) from healthy controls (Table 1).

**Table 1:** Characteristics of participants.

Characteristics	TB (N = 58)	Control (N = 32)
Male/female	38/20	21/11
Age, mean (range) years	40.83±18.04 (12–70)	37.53±15.16 (15–62)
TST test	Not applicable	Negative
Both sputum smear and culture positive	32	0
Smear negative while culture positive	26	0
Cough	46	0
Fever	42	0
Weight loss	39	0
Night sweats	32	0
Hemoptysis	27	0

All patients had clinical signs and symptoms of active pulmonary TB; comprising, 79.3% cough, 72.4% fever, 67.2% weight loss, 55.2% night sweats, and 46.6% hemoptysis. Healthy controls involved in the study, were free of active TB infection, latent TB infection and any clinical symptoms of any infectious disease. Both TB patients and healthy controls were non-smokers.

**Table 2:** Oligonucleotides used in this study.

Primer set name	Reverse transcriptase reaction primer (5′ to 3′)	Real-time quantitative PCR primer (5′ to 3′)
U6	CGCTTCACGAATTTGCGTGTCAT	Forward: GCTTCGGCAGCACATATACTAAAAT Reverse: CGCTTCACGAATTTGCGTGTCAT
hsa-miR- 19b-2*	GTCGTATCCAGTGCGTGTCGTGGAGTCGGCAATTGCACTGGATACGACTGAAATG	Forward: GGGAGTTTTGCAGGTTTG Reverse: CAGTGCGTGTCGTGGA
hsa-miR- 3179	GTCGTATCCAGTGCGTGTCGTGGAGTCGGCAATTGCACTGGATACGACACGTTTA	Forward: GGGAGAAGGGGTGAAAT Reverse: CAGTGCGTGTCGTGGA
hsa-miR- 147	GTCGTATCCAGTGCGTGTCGTGGAGTCG GCAATTGCACTGGATACGACGCAGAAG	Forward: GGGGTGTGTGGAAATG Reverse: CAGTGCGTGTCGTGG

The study was approved by the ethics committee of Weifang Medical University and was carried out in compliance with the Helsinki Declaration. Informed consent was obtained from all subjects prior to beginning the study.

### Sample preparation, RNA isolation and RNA quality control

Early morning sputum samples, a minimum of 1.5 ml, were collected in a sterile, disposable plastic containers before starting chemotherapy [16] and were then solubilized with an equal volume of 0.1% dithiothreitol within 1 h of collection. Samples were placed at 37°C for 30 min to ensure complete homogenization, and were subsequently centrifuged to yield cell free supernatant and cell pellet. To exclude salivary contamination, pellet cells were stained with Wright's stain for differential cell counts. A sputum sample was considered adequate for further analysis when percentage of squamous cells was less than 80% [17], [18], [19]. Cell free supernatant was aliquoted and stored immediately in liquid nitrogen until analysis.

**Figure 1:**
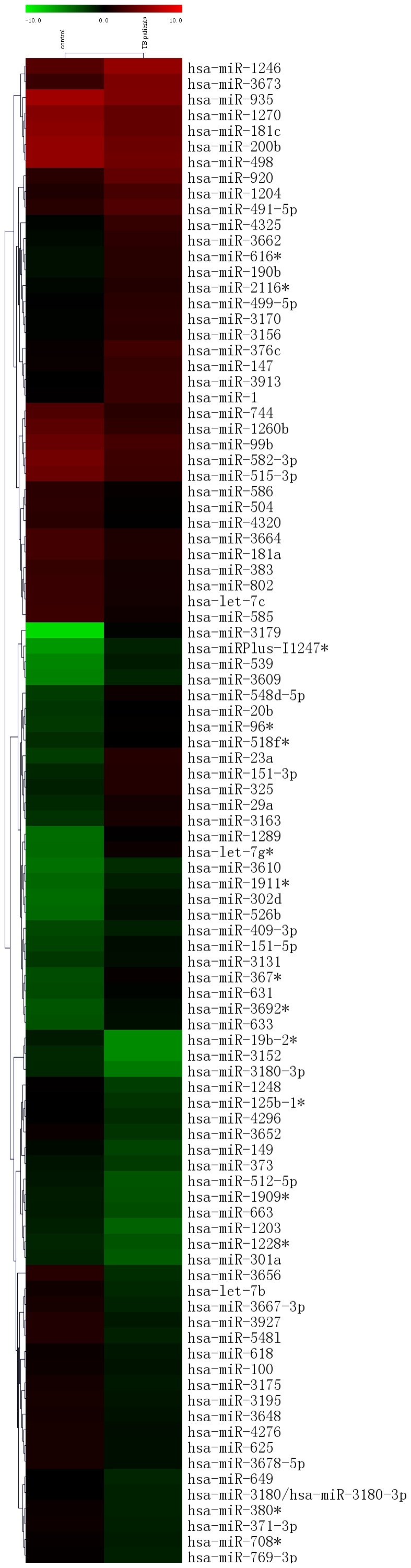
Hierarchical clustering of miRNA in sputum samples. Samples were clustered according to the signature profile of 97 differentially expressed miRNAs. Data from each miRNA were median centered. Samples were in columns and miRNAs in rows. Red and green indicated high relative level and low relative level, respectively. The *P* values for these miRNAs were less than 0.05 in TB group compared with controls.

**Table 3:** Overexpressed miRNAs in TB sputum.

Name	TB patients versus control	Chromosome
hsa-miR-2116*	3.08	15
hsa-miR-409-3p	3.09	14
hsa-miR-1204	3.10	8
hsa-miR-3131	3.15	2
hsa-miR-491-5p	3.20	9
hsa-miR-499-5p	3.22	20
hsa-miR-147	3.34	9
hsa-miR-518f*	3.40	19
hsa-miR-3156	3.45	10
hsa-miR-3170	3.63	13
hsa-miR-20b	4.18	X
hsa-miR-151-5p	4.29	8
hsa-miR-1	4.44	20
hsa-miR-920	4.47	12
hsa-miR-3913	4.48	12
hsa-miR-190b	4.50	1
hsa-miR-616*	4.54	12
hsa-miR-3662	4.61	6
hsa-miR-376c	4.75	14
hsa-miR-4325	4.85	20
hsa-miR-29a	5.21	7
hsa-miR-96*	5.24	7
hsa-miR-1246	5.26	2
hsa-miR-325	5.96	X
hsa-miR-3610	6.27	8
hsa-miR-633	6.27	17
hsa-miR-3673	6.34	8
hsa-miR-631	6.71	15
hsa-miR-1911*	7.03	X
hsa-miR-548d-5p	7.32	17
hsa-miR-3692*	7.54	6
hsa-miR-151-3p	7.60	8
hsa-miR-3163	7.65	11
hsa-miR-367*	10.1	4
hsa-miR-302d	11.7	4
hsa-miR-526b	11.9	19
hsa-miR-3609	13.1	7
hsa-miR-23a	14.2	19
hsa-miR-539	18.2	14
hsa-miR-1289	20.8	20
hsa-miRPlus-I1247*	24.4	Unknown
hsa-let-7g*	25.8	3
hsa-miR-3179	347	16

Column “Name” contained the name of miRNA; Column “TB patients versus control” contained level ratio of TB/control; Column “Chromosome” meant distribution of each miRNA on chromosome.

Each miRNA spot was replicated for four times on the same slide and two microarray chips were used for each group. After normalization, obtained average values for each miRNA spot were used for statistics. The *P* values for these miRNAs were less than 0.05 in TB group compared with controls.

Total RNA was extracted using Trizol reagent (Invitrogen) and further purified with a RNeasy mini kit (Qiagen, Denmark) according to the manufacturer's instructions. RNA quantity and quality were assessed using NanoDrop Specthophotometer (ND-1000, Nanodrop Technologies) and electrophoresis, respectively [20]. RNA concentrations ranged from 51 to 67 ng/μl. Equal amounts (300 ng) of RNA from each patient and control were pooled and presented as two groups (TB and control), respectively. MiRNA signature profiles were generated from the above two groups.

**Table 4:** Underexpressed miRNAs in TB sputum.

Name	TB patients versus control	Chromosome
hsa-miR-19b-2*	0.04	X
hsa-miR-3152	0.06	9
hsa-miR-3656	0.09	11
hsa-miR-3180-3p	0.10	16
hsa-miR-1203	0.15	17
hsa-miR-548l	0.16	11
hsa-miR-1248	0.17	3
hsa-miR-3652	0.18	12
hsa-miR-512-5p	0.19	19
hsa-miR-3667-3p	0.20	22
hsa-miR-149	0.20	2
hsa-miR-3927	0.20	9
hsa-let-7b	0.21	22
hsa-miR-301a	0.21	17
hsa-miR-1909*	0.22	19
hsa-miR-125b-1*	0.22	11
hsa-miR-582-3p	0.23	5
hsa-miR-663	0.24	20
hsa-miR-515-3p	0.26	19
hsa-miR-1228*	0.27	12
hsa-miR-4296	0.28	10
hsa-miR-3195	0.29	20
hsa-miR-1260b	0.29	11
hsa-miR-380*	0.29	14
hsa-miR-3175	0.29	15
hsa-miR-371-3p	0.30	19
hsa-miR-585	0.30	5
hsa-miR-4320	0.31	18
hsa-miR-504	0.32	X
hsa-miR-200b	0.33	1
hsa-miR-181c	0.33	19
hsa-let-7c	0.34	21
hsa-miR-4276	0.34	4
hsa-miR-625	0.35	14
hsa-miR-3664	0.35	11
hsa-miR-3648	0.35	21
hsa-miR-373	0.35	19
hsa-miR-3678-5p	0.35	17
hsa-miR-744	0.36	17
hsa-miR-769-3p	0.36	19
hsa-miR-649	0.36	22
hsa-miR-586	0.37	6
hsa-miR-498	0.38	19
hsa-miR-708*	0.38	11
hsa-miR-99b	0.38	19
hsa-miR-935	0.38	19
hsa-miR-802	0.38	21
hsa-miR-618	0.38	12
hsa-miR-181a	0.38	9
hsa-miR-1270	0.39	19
hsa-miR-100	0.39	11
hsa-miR-383	0.39	8

Column “Name” contained the name of miRNA; Column “TB patients versus control” contained level ratio of TB/control; Column “Chromosome” meant distribution of each miRNA on chromosome.

Each miRNA spot was replicated for four times on the same slide and two microarray chips were used for each group. After normalization, obtained average values for each miRNA spot were used for statistics. The *P* values for these miRNAs were less than 0.05 in TB group compared with controls.

**Figure 2:**
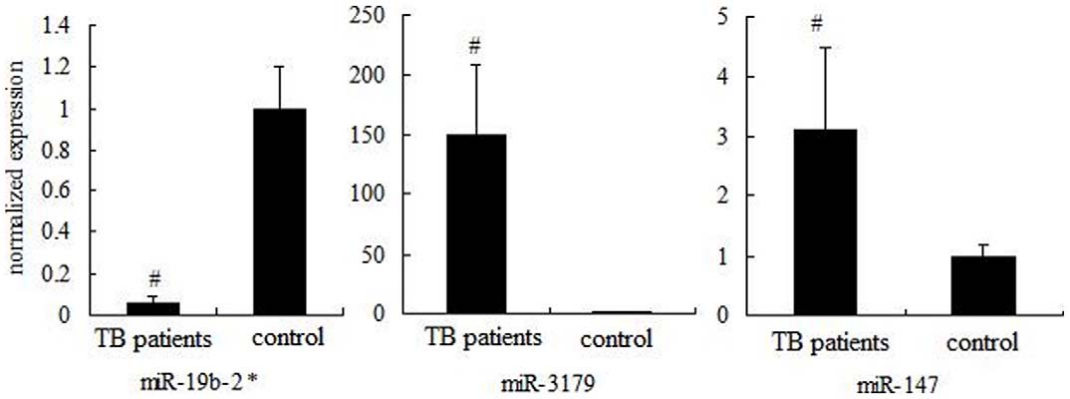
Confirmation miRNA level by RT-qPCR. RT-qPCR analysis confirmed microarray data. After normalization to U6 RNA, data were presented as mean ± SD (n = 30) and obtained average value for each miRNA was used for statistics. MiR-19b-2* was underexpressed while miR-3179 and miR-147 were overexpressed in TB sputum compared with controls. The experiment was conducted in triplicate. ^#^ *P*<0.05 versus control.

### Detection of miRNAs expression and data analysis

Exiqon miRCURY^TM^ LNA arrays v.16.0, containing ∼1223 capture probes covering all human, were used to quantify genome-wide miRNA expression in the two groups described above. One microgram of each group was 3′-end-labeled with a miRCURYTM Hy3TM power labeling kit (Exiqon, Vedbaek) and hybridized on the LNA arrays according to the manufacturer's instructions. Images on the chip were scanned using an Axon GenePix 4000B microarray scanner (Axon Instruments, Foster City, CA) and imported into GenePix Pro 6.0 software (Axon) for grid alignment and data extraction. MiRNAs with intensities >50 were used to calculate the normalization factor. Expression data were normalized using the median normalization. After normalization, average values of replicate spots of each miRNA were used for statistical analysis. Hierarchical clustering was performed using MEV software (v4.6, TIGR).

### RT-qPCR analysis

To validate the microarray results, RT-qPCR was further performed using individual samples from a randomly selected subgroup (30 patients with active pulmonary TB and 30 healthy controls).

miR-19b-2*, miR-3179 and miR-147 were selected for RT-qPCR analysis. First-strand cDNA was generated from ∼200 ng of total RNA using miRNA-specific primers (Table 2). Approximately 2.5 ng of cDNA was then used for PCR analysis using a GeneAmp PCR System 9700 (Applied Biosystems, USA). The threshold cycle (Ct) is defined as the fractional cycle number at which the fluorescence passes the fixed threshold. Each miRNA level was normalized with U6 small nuclear RNA level. All samples were run in triplicate and repeated a minimum of two times.

### Enzyme-linked immunosorbent assay (ELISA) analysis

Sputum samples were collected as described above. Protease inhibitors were used to prevent proteolytic degradation during sputum solubilization. Cytokine levels were measured from 50 μl of cell-free supernatants using commercially available ELISA kits (BioSource, Nivelles, Belgium) according to the manufacturer's instructions. All samples were assayed in duplicate.

### Statistical analysis

Data were presented as mean ± standard deviation (SD). ANOVA test or student's *t* test was used for statistical analysis. *P*<0.05 was regarded as significantly different.

## Results

### Differential expression of miRNAs between TB patients and controls

A total of 95 miRNAs were differentially expressed between TB group and controls. Forty-three miRNAs were overexpressed and fifty-two miRNAs were underexpressed in TB group compared with controls. MiRNAs levels in TB group were 3.08- to 340- fold overexpression, but 2.56- to 25.0- fold underexpression in comparison with controls (Tables 3, 4), respectively. Cluster analysis based on these differentially expressed miRNAs showed a clear distinction between TB group and controls (Figure 1).

### Validation of microarray results by RT-qPCR

Three miRNAs, miR-19b-2*, miR-3179 and miR-147, were selected for further validation of microarray results using RT-qPCR. The reasons for choosing them were that miR-19b-2* and miR-3179 were the most underexpressed and overexpressed miRNAs in TB group compared with controls, respectively. MiR-147 was of an extra interest because it is a negative regulator of inflammatory responses [8]. To better understand the association between TB infection and miR-147, it was selected for validation.

Data analysis showed that miR-19b-2* was underexpressed while miR-3179 and miR-147 were overexpressed in TB group compared with controls. The RT-qPCR results were consistent with those of microarray (Figure 2).

### Measurement of cytokines levels

Both microarray and RT-qPCR results showed that miR-147 was overexpressed in TB group compared with controls. Considering the role of miR-147 in reduction of TNF-α and IL-6 [8], we hypothesized that TNF-α and IL-6 levels in TB sputum might not be altered. Consistent with the hypothesis, their levels were not obviously alterated between TB group and controls (data not shown).

## Discussion

Recent studies have shown that infection of human macrophages with *Mycobacterium avium hominissuis* causes a specific miRNA response [21] and infection of mice with *Mycobacterium bovis bacillus Calmette-Guérin* (BCG) downregulates miR-29 expression [22]. These findings open up new and interesting avenues for an improved understanding of the link between miRNAs homeostasis and TB infection.

In this study, differentially expressed 95 miRNAs were identified between TB sputum and controls. In our previous study, 92 miRNAs with differential expression were identified between TB serum and controls [23]. It is worth mentioning that the results from TB sputum were largely inconsistent with those from TB serum. For example, miR-19b-2* and miR-3179 were the most underexpressed and overexpressed miRNAs in TB sputum compared with controls, respectively. However, in comparison with controls, the most underexpressed and overexpressed miRNAs in TB serum were miR-518d-5p and miR-93*, respectively. The detailed mechanism for the difference is unknown.

In the study, functions of most differentially expressed miRNAs are still largely unknown, such as miR-19b-2* and miR-3179. However, some miRNAs, such as miR-301a, miR-373 and miR-let-7, have been functionally linked to specific signaling pathways. MiR-301a overexpression contributes to NF-κB activation in pancreatic cancer cells [24]. MiR-373 promotes tumour invasion and metastasis [25]. Our results indicate an additional mode of action for these important miRNAs. Recent studies have shown that miR-let-7b may contribute significantly to the regulation of IFN-β in innate immune response [26] and miR-let-7c is underexpressed in sputum of patients with chronic obstructive pulmonary disease [14]. Our results showed that miR-let-7b and miR-let-7c were underexpressed while miR-let-7g* was overexpressed in TB sputum compared with controls, which suggests that let-7 family members are also involved in regulation of anti-TB immune response.

It is worth mentioning that several miRNAs, such as miR-150 and miR-155 [27], [28], have been shown to regulate adaptive immune response, while our results showed that their levels in both sputum and serum were not changed significantly between TB group and controls, which suggests that other miRNAs may be involved in regulation of anti-TB immune response.

A recent study shows that miR-29a is specifically overexpressed after mycobacterial infection of human macrophages [21], which is in good consistency with our results that miR-29a was 5.21- and 11.9-fold overexpression in TB sputum and serum compared with controls, respectively. Interferon-γ (IFN-γ) has a critical role in immune responses to intracellular bacterial infection and miR-29 is found to suppress immune responses to intracellular pathogens by downregulating IFN-γ [22]. Overexpressed miR-29a in our study could partly explain one mechanism by which *M. tuberculosis* avoids macrophage killing through inhibition of IFN-γ-mediated signaling. These data suggest that miR-29a might act as a negative regulator of immune response against TB infection.

The preferred niche of *M. tuberculosis* is the macrophages [29]. Macrophages recognize invading *M. tuberculosis* primarily through Toll-like receptors (TLRs) [30]. TLR signals can activate NF-κB, which results in induction of multiple pro-inflammatory cytokines, suca as TNF-α and IL-6 [31]. A recent study shows that, upon activation of TLRs/NF-κB signaling pathway, miR-147 is induced in murine macrophages and attenuates expression of proinflammatory cytokines, such as TNF-α and IL-6, which indicates that miR-147 appears to have potent anti-inflammatory properties [8]. Our results showed that miR-147 was 3.34-fold overexpression in TB sputum compared with controls. Considering the role of miR-147 in down-regulating excessive inflammatory responses, we hypothesized that both TNF-α and IL-6 levels in TB sputum are not overexpressed in comparison with controls. Consistent with the hypothesis, levels of TNF-α and IL-6 did not differ significantly between TB group and controls. MiR-147 attenuates TLRs stimulation-induced-inflammatory response, however, the detailed mechanism is unknown. Targets for miR-147 identified by computational prediction programs, such as TargetScan, have not previously been shown to be directly involved in TLR signaling events. Thus, the mechanism by which miR-147 regulates TLR-induced inflammatory responses requires further investigation.

Taken together, we showed for the first time that miRNA expression profiles in sputum were significantly altered during TB infection, which provides rationale for studying the role of miRNAs in the pathogenesis of active pulmonary TB and indicates potential for improving diagnosis, prognosis and their impact on future therapeutic strategies. However, it is difficult to synthesize the study results to reach a definitive conclusion based on this single study. The association between TB infection and miRNAs is just beginning to be explored and further investigation is required to determine the role of miRNAs in active pulmonary tuberculosis.
